# Supplementary material for: Aptamer against Aflatoxin B1 Obtained by SELEX and Applied in Detection
Source: Biosensors (Basel). 2022 Oct 9;12(10):848. doi: 10.3390/bios12100848 (PMC9599246; doi:10.3390/bios12100848)
Supplement: Supplementary file 1 [file biosensors-12-00848-s001.zip › biosensors-1941106-supplementary.pdf]

A

NH_2-MNPs (μg)	25	50	100	125	Control (no beads)
AFB_1 input (ppb)	2.803	2.803	2.803	2.803	2.803
AFB_1 on MNPs (ppb)	1.207	0.32	0.9	0.396	-
Relative yield (%)	43	11	32	14	-

B

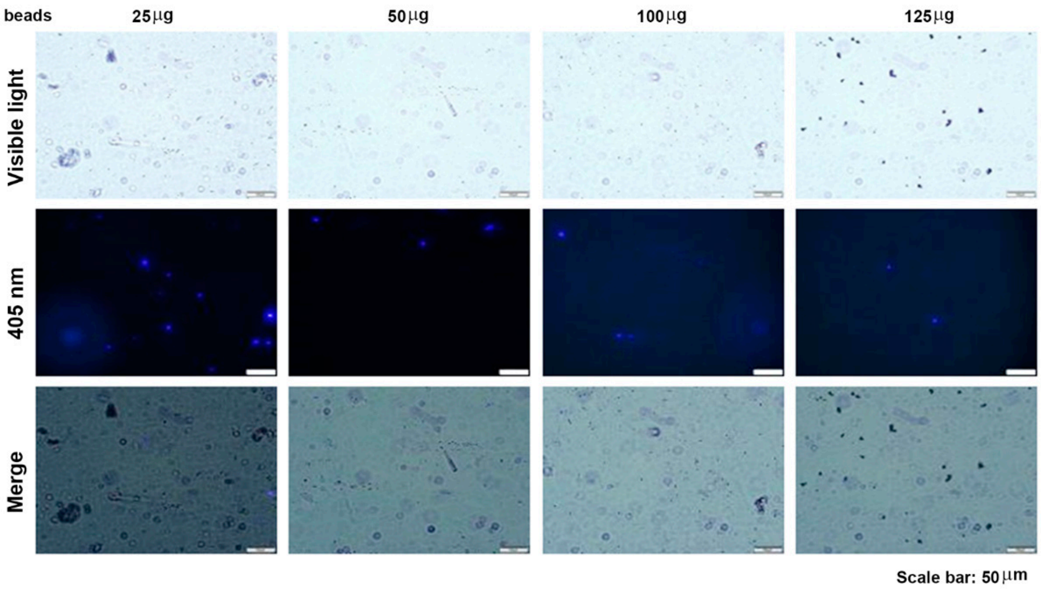

Figure S1. Conjugation efficiency and fluorescent image of AFB1 linked to magnetic nanoparticle (NMP) beads. (A) Conjugation of AFB1 linked to the NMP beads. (B) Images of the fluorescent signal derived from the AFB1-linked beads.

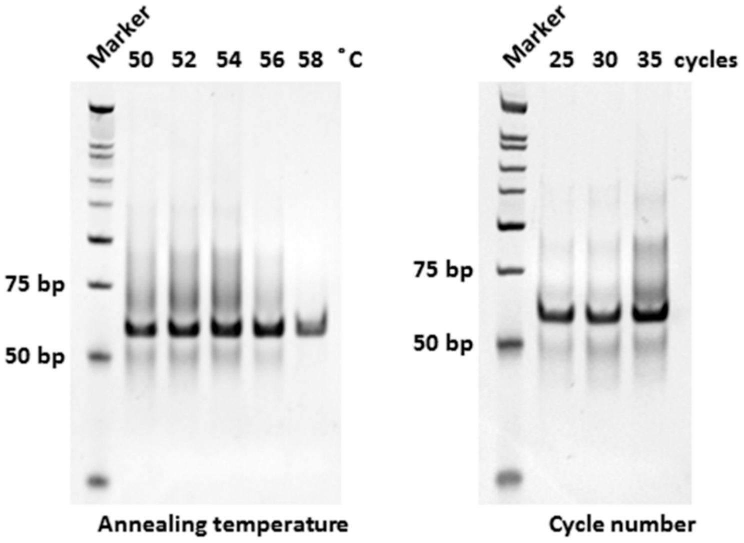

Figure S2. Optimization of PCR conditions to obtain consistent and correct size-specific products.

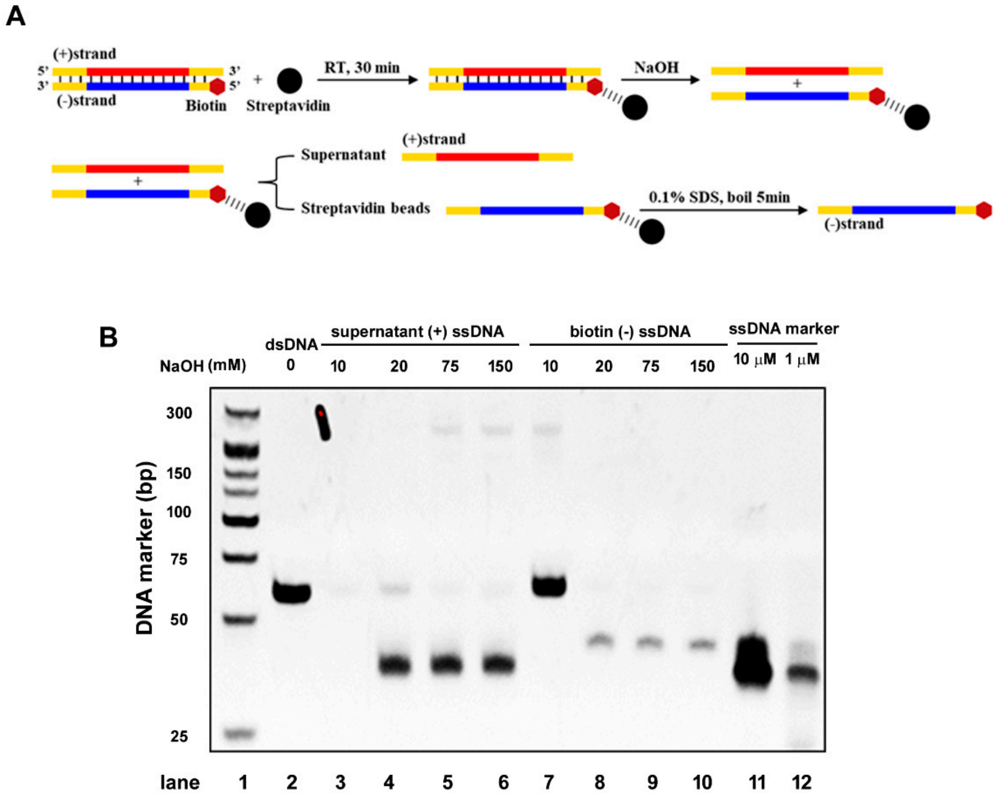

Figure S3. Strategy of obtaining single-strand DNA from the PCR products. (A) Schematic illustration of obtaining the single-strand DNA from PCR products. (B) Optimization of ssDNA isolation with various concentrations of NaOH treatment. Lane 1: DNA marker. Lane 2: dsDNA isolated from SELEX cycle (57 bp). Lane 3–6: the (+)ssDNA isolated from the PCR products with the alkaline treatment as indicated, Lane 7–10: the (-)ssDNA isolated from streptavidin-linked Dynabeads with the alkaline treatment as indicated, Lane 11–12: 10 and 1 μM ssDNA marker (57 bases), respectively.

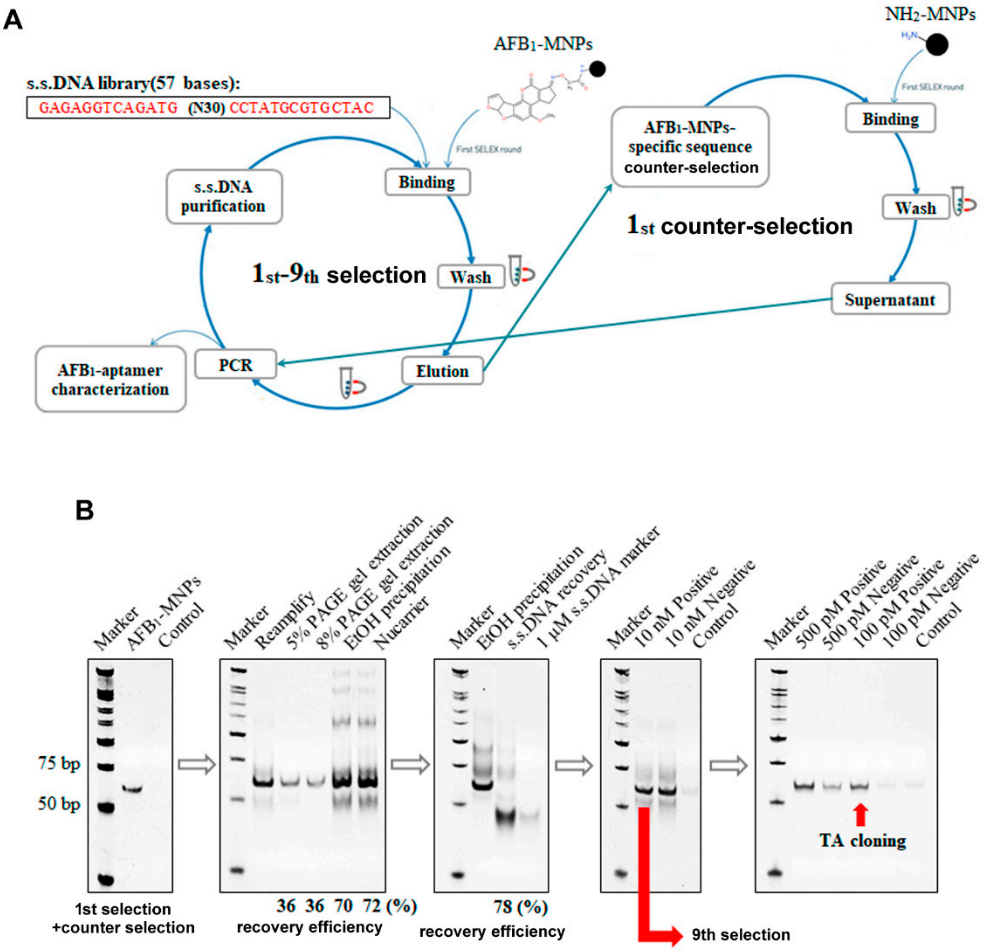

Figure S4. Process of SELEX technology. (A) Schematic illustration of the SELEX process including the positive selection and the counter-selection. (B) Analysis of the selected PCR products and their recovery efficiency from polyacrylamide gel (5% or 8% indicated). After the 9th round of selection, the positive selection product was used for TA-vector cloning as indicated.

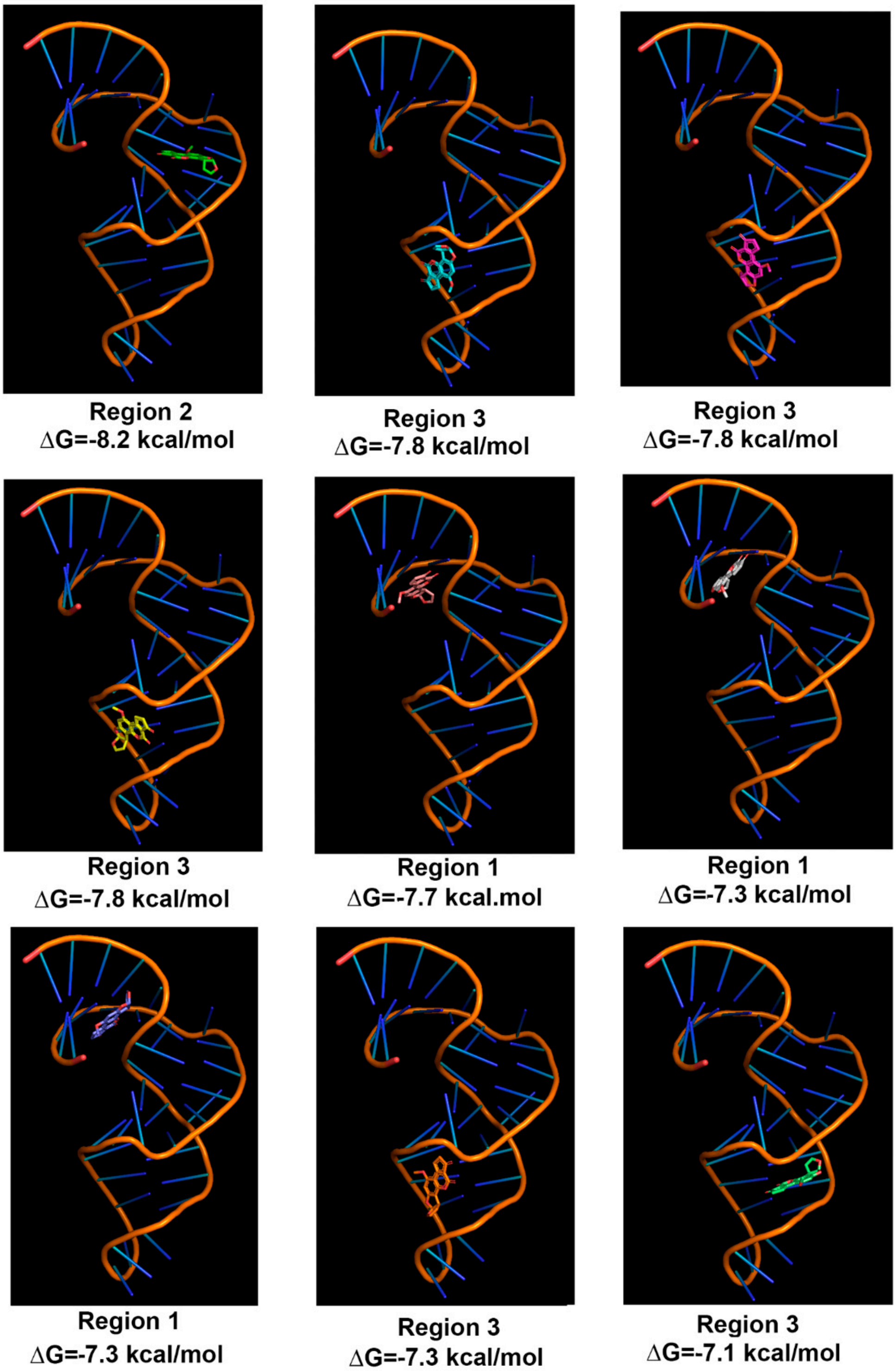

Figure S5. Nine possible 3D structural models of the interaction between fl−2CS1/core and AFB1. Location of the docking site and the Δ*G* of these interactions is indicated.
